# Self-assembled polylactic acid (PLA): Synthesis, properties and biomedical applications

**DOI:** 10.3389/fchem.2022.1107620

**Published:** 2023-01-06

**Authors:** Tianyu Chen, Xiaoying Zhao, Yunxuan Weng

**Affiliations:** ^1^ College of Chemistry and Materials Engineering, Beijing Technology and Business University, Beijing, China; ^2^ Beijing Key Laboratory of Quality Evaluation Technology for Hygiene and Safety of Plastics, Beijing Technology and Business University, Beijing, China

**Keywords:** self-assembly, polylactic acid, surface microstructure, surface topography, biomaterials

## Abstract

The surface morphology and topography of cell culture substrates play an important role in cell proliferation and growth. Regulation of the surface microstructure allows the development of tissue culture media suitable for different cells. Polylactic acid (PLA) is a biobased and biodegradable (under defined conditions) polymer with low immunogenicity, non-toxicity, and good mechanical properties, which have facilitated their pharmaceutical and biomedical applications. This review summarizes recent advances in the synthesis and self-assembly of surface microstructure based on PLA materials and discusses their biomedical applications such as cell culturing and tissue engineering.

## 1 Introduction

Self-assembly is a bottom-up strategy to fabricate nano- and microstructures with novel properties. It is simple, economical, precise, flexible and has been widely used to construct polymers with advanced structures and biomedical applications (Qi et al., 2018). In the biomedical field, the surface topography of the biomaterials affect its interaction with the cells and determines the orientation of cell growth, a phenomenon known as the “contact guidance” effect (Weiss, 1968; Ermis et al., 2018). The cell-biomaterial interaction involves in the mutual molecular recognition between the receptors on cell surface and the corresponding ligands from the biomaterials (van Kooten et al., 2004). In addition, the topological microstructure of the biomaterials provides high surface volume ratio which can enrich nutrient absorption and promote cell adhesion and growth (Mi et al., 2013; Wu et al., 2014; Wang et al., 2016). To summarize, the surface topography of the biomaterials has a great influence on the adhesion, spreading, proliferation, and functional expression of cells. In order to fabricate different microstructures, various kinds of manufacturing techniques have been developed, such as 3D print (Zhou et al., 2016; Wu et al., 2020), plasma etching (da Silva and Rosa, 2022; Ozaltin et al., 2022), lithography (Jeong et al., 2015; Sun et al., 2016), *etc.* However, aforesaid technologies are complex and precise control of the nanometer-sized features is still difficult to be achieved. This review discusses the techniques of preparing self-assembled polylactic acid (PLA) with microstructured surfaces, including their unique properties especially the topographical properties and their application in the biomedical field, such as cell culture substrates, transplant scaffolds, drug-controlled release, wound dressings, *etc.*

## 2 Biomedical applications of PLA with microstructured surfaces

**FIGURE 1:**
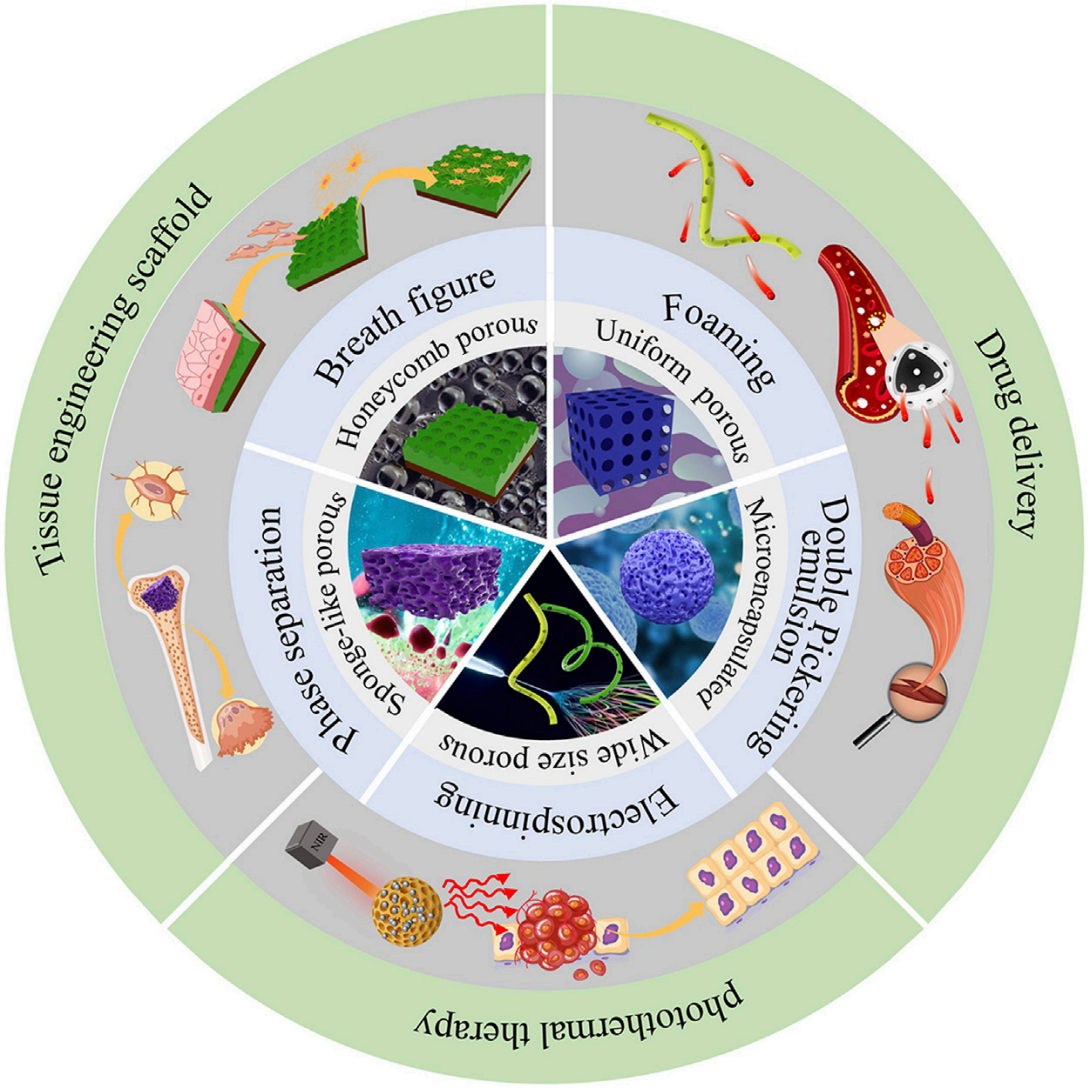
Fabrication methods and biomedical applications of self-assembled PLA.

PLA materials fabricated with self-assembly strategies with specific surface topographies have been increasingly used in biomedical field (Figure 1). For example, for tissue engineering applications, highly porous polymer matrices are needed to provide a homogenously distributed cell seeding density and effective oxygen and nutrient supply to maintain cell viability (Shah Mohammadi et al., 2014). Their high specific surface area can also provide cells with a large space per unit volume for cell adhesion (Mi et al., 2013). Therefore, PLA with porous surface topographies has been used as templates for tissue regeneration, to provide sufficient internal space to promote cell division and growth, as well as the transportation of nutrients and oxygen and the excretion of metabolic waste (Wang et al., 2013; Kuang et al., 2017).

Another biomedical applications of PLA with self-assembled surface topography include carriers and sustained release of drugs (Liu et al., 2020). Biodegradable PLA can be used as a drug carrier to control the drug release rate by controlling PLA degradation rate. The use of PLA as a drug carrier can avoid secondary damage caused by the removal of non-degradable substrates (Riley et al., 2003; Lassalle and Ferreira, 2007). Sustained and targeted drug release can also be achieved *via* microcapsulation technique (Bysell et al., 2011). Han et al. fabricated a exosomes BMP-2 containing microcapsule using polyaspartic acidpolylactic acid-glycolic acid copolymer (PASP-PLGA) for controlled drug release to promote tendon bone healing (Han et al., 2022). Microemulsion technique has also been used to capsulate hydrophilic drugs using PLA (Zhu et al., 2018).

PLA microcapsule can also be used for tumor-targeting and tumor photothermal therapy (Liu et al., 2020). Jin et al. introduced gold nanoparticles into PLA microcapsules through double-microemulsion technique, followed by depositing graphene oxide onto the microcapsule surface *via* electrostatic layer-by-layer self-assembly technique. Therapeutic diagnostic microcapsules were formed upon the solvent evaporation. With the near-infrared laser light irradiation for 9 days, the tumor was ablated completely and the tumor growth inhibition was 83.8% in the presence of the microcapsules (Jin et al., 2013).

## 3 PLA with microstructured surfaces—Preparation methods and properties

### 3.1 Breath figure (BF)

Breath figure (BF) is a phenomenon of water vapor comes into contact with cold surface (solid or liquid) and self-assembly, forming a set of fog droplets (Bormashenko, 2017). BF allows the fabrication of well-controlled microporous topography by rapidly evaporating polymer solutions in a humid atmosphere (Widawski et al., 1994; François et al., 1995; Pitois and François, 1999). Francois first reported the construction of honeycomb (HC) structured polymer films by BF in 1994 (Widawski et al., 1994). Since then, BF has attracted great research interest in fabricating polymer materials with microporous surface due to its simplicity, low cost, robust mechanism of pattern formation and flexibility of pattern tailoring (Bunz, 2006; Stenzel et al., 2006; Mansouri et al., 2013; Zhang et al., 2015).

**FIGURE 2:**
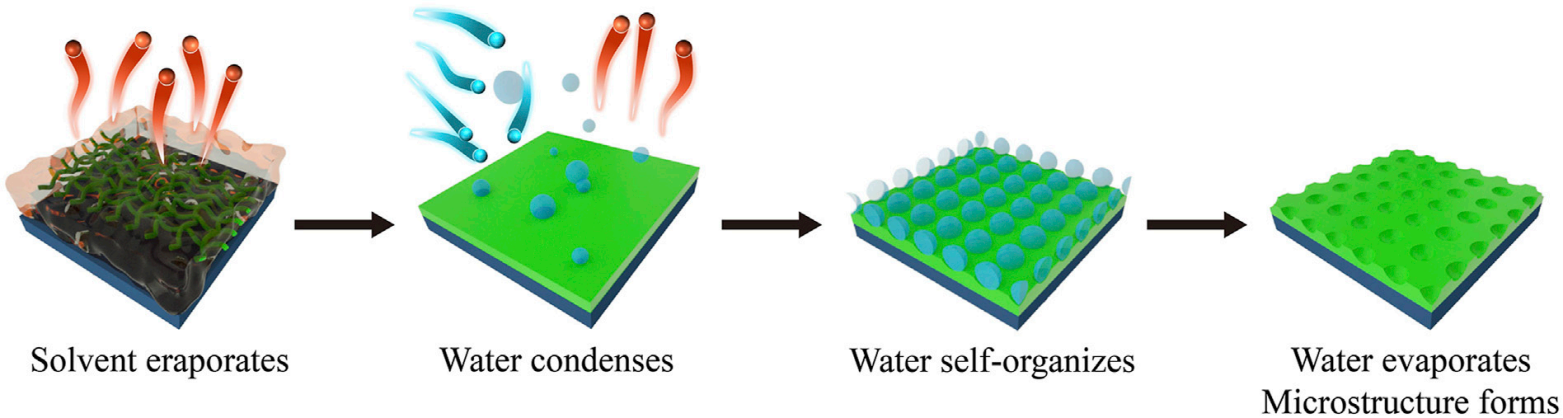
Fabrication of honeycomb patterns using breath figure technique.

The construction of polymer materials with HC morphology mainly include three steps (Huang et al., 2014): i) dissolve the polymer in a good organic solvent and cast the solution on a solid substrate. The organic solvent rapidly evaporates, decreasing the surface temperature of the solution from room temperature to near 0°C; ii) the water vapor in the air quickly nucleates and condenses on the cold surface of the solution to form small droplets; iii) the water droplets are assembled under the action of capillary force to form hexagonal arranged micropores with sizes ranging from hundreds of nanometers to several microns. As a result, polymer films with honeycomb-patterned microporous surface are fabricated with one step using BF (Figure 2).

Polymer films with honeycomb-patterned topography can enhance cell adhesion, spreading, proliferation, and differentiation. Wu et al. studied honeycomb-patterned films incorporated with other components for osteoblast cultivation (Wu et al., 2014). They fabricated honeycomb poly (l-lactide) (PLLA) films incorporated with nano-hydroxyapatite (nHA) (3 wt%, 5 wt%, and 7 wt%) and evaluated the effect of nHA on the self-assembly of honeycomb patterns on PLLA films. In order to investigate the effect of the honeycomb pattern and nHA on the cell, MC3T3-E1 mouse newborn calvaria preosteoblasts were cultured on the film. The porous structure on films effectively increased the surface contact area with proteins, resulting in enhanced serum and fibronectin protein adsorptions and accelerated cell proliferation. The honeycomb pores also enhanced cell differentiation, especially when incorporated with nHA, which can enhance the interactions between the cells and the serum protein and promote osteoblast adhesion. Yin et al. developed hydrophilic honeycomb-patterned PLA films *via* BF with the assistance of ionic surfactant dodecyltrimethylammonium chloride (DTAC) (Yin et al., 2019). DTAC was preferentially oriented at the solution-air interface with its cationic hydrophilic head group in the air. As a result, the interfacial tension between the solution and water was decreased, which can help stabilize the condensed water droplets during the BF process, leading to the formation of highly ordered honeycomb pattern and enhancement of PLA surface wettability. The surface morphology of the film can be readily regulated by adjusting the ratio of PLA to DTAC. The hydrophilic PLA films with honeycomb patterns can promote cell attachment when used as a scaffold and outperformed the regularly used cell adhesion material Poly-l-lysine (PLL) (Yin et al., 2019).

#### 3.1.1 Reverse breath figure (RBF)

The mechanism of RBF is the same as that of BF, but its process of pattern formation is different than BF. During RBF, a saturated organic non-solvent vapor environment with a specific level of humidity is first prepared, then the water in the environment condenses on the surface of a substrate (e.g., Petri dish), forming a layer of water-organic mixed droplets. Then a polymer solution is casted on the substrate, and after the solvent evaporates, a polymer film with a surface having a microsphere pattern is formed (Xiong et al., 2009). As this process is opposite to BF, which involves in casting the polymer solution first and condensing the water droplets afterwards, the surface pattern obtained from RBF is a microsphere structure where that from BF is a honeycomb porous structure (Duarte et al., 2017). The diameter of the microstructure formed by both BF and RBF ranges from hundreds of nanometers to several micrometers (Ferrari et al., 2011).

The dense microsphere structure on the surface of the polymer film from RBF can facilitate the extension and growth of cells (Duarte et al., 2017). Duarte et al. fabricated a microsphere surface film containing starch and poly-lactic acid (SPLA) using RBF (Duarte et al., 2017). *In vitro* studies showed that the cells had a good interaction with the film after 24 h in culture (Duarte et al., 2017).

### 3.2 Phase separation

Phase-separation technique can be applied to produce 3-D porous materials. This process is based on the inducement of thermal treatment, which can lower the free energy of a homogeneous polymer solution and make it thermodynamically unstable and tend to separate into a continuous multiphase system (Zhou et al., 2012). The multiphase system consists of two phases, one with a high polymer concentration, i.e., polymer-rich phase and another one with a low polymer concentration, i.e., polymer-lean phase. Either the former or the later will separate from the solution as a dispersed phase. Upon the solvent evaporation, the polymer-rich phase solidifies to transform into a sponge-like structure, while the polymer-lean phase becomes a porous membrane (van de Witte et al., 1996; Guillen et al., 2011). The key of using phase separation method to fabricate self-assembled polymer materials is changing the Gibbs free energy in the polymer solution systems through certain ways (Kamide et al., 1993; Ismail et al., 2020). Based on the difference in the thermodynamic state of the polymer solution, the phase separation method is mainly divided into non-solvent induced phase separation (NIPS), thermally induced phase separation (TIPS) and vapour induced phase separation (VIPS) (Kim et al., 2016).

#### 3.2.1 Non-solvent induced phase separation (NIPS)

Dispersing non-solvents in a stable polymer solution will change the free energy of the system and the system will change from a compatible state to a substable and incompatible state, leading to the formation of a two-phase structure with the polymer as the continuous phase and the solvent as the dispersed phase on the surface (Huang and Thomas, 2018). This process can obtain a microporous structure up on the solvent evaporation (Rezabeigi and Demarquette, 2019). Liquid-liquid phase separation and solid-liquid phase separation are the two main types of phase separation. The former forms a polymer-rich phase and a polymer-lean phase, and the latter forms a polymer-lean phase and a polymer precipitate phase, which leads to the occurrence of liquid-liquid phase separation in the system. Liquid-liquid phase separation can be used to construct porous structures of PLA. Polymer-solvent-non-solvent systems have specific phase separation behavior and kinetics, i.e., changes in the ratio of the non-solvent to solvent can affect the interaction between the polymer and solvent and promotes changes in Gibbs free energy, leading to changes in the porous structures of the polymers (Garcia et al., 2020).

Bui et al. fabricated PLA honeycomb films with controllable pore dimensions using NIPS method (Bui et al., 2017). They prepared a ternary polymer-solvent-nonsolvent PLA solution using methanol and chloroform as bad and good solvent, respectively. Phase separation occurred in the system and resulted in the formation of ordered honeycomb pattern. The films were used as a scaffold to culture NIH3T3 cells. The effect of the film surface topography on cell adhesion, proliferation, and viability were systematically investigated. Compared with the unmodified PLA film, the cell density significantly increased on the honeycomb-patterned films which had an average pore size of approximately 6 μm. They concluded that the porous structure was highly favored as it could promote nutrient supply and provide anchorage points to facilitate tight cell adhesion to the culture (Bui et al., 2017).

#### 3.2.2 Thermally induced phase separation (TIPS)

Thermally induced phase separation occurs by rapidly cooling the polymer solution or evaporating the solvent to induce phase separation by forming a polymer-rich and a solvent-rich phase (Szewczyk and Stachewicz, 2020). A porous structure is obtained after the solvent evaporation is completed (Akbarzadeh and Yousefi, 2014). TIPS has been used to assist electrospinning to develop microporous PLA fibers. Honarbakhsh et al. fabricated drug delivery scaffolds with PLA/poly (ethylene oxide) (PEO) blends using TIPS and electrospinning technique (Honarbakhsh and Pourdeyhimi, 2011). The solution of the polymer blends (Dichloromethane solvent) was first electrospun and then underwent TIPS upon solvent evaporation, leading to the formation of microporous fibers. The presence of the hydrophilic (PEO) and hydrophobic (PLA) segments in the structure can help reduce post-implantation complications such as platelet adhesion. The enhanced hydrophilicity of PLA can also help provide preferable sites for the attachment of the aqueous compounds without interfering the structural integrity and porous morphology of the fibers. Moreover, the porous structure of the fibers, owing to their large specific surface area coupled with high porosity, can facilitate drug diffusion and improve the fluid transport (Honarbakhsh and Pourdeyhimi, 2011).

#### 3.2.3 Vapour induced phase separation (VIPS)

Vapour induced phase separation occurs in a humid environment, by absorbing non-solvent vapor (water in most cases) from the ambient air (Wang and Lai, 2013; Xu et al., 2020). The polymer solution is subjected to non-solvent vapor, and phase separation begins when the non-solvent in the environment penetrates the solution. Micropores on the surface of the polymer film are obtained after the solvent is evaporated (Wang and Lai, 2013; Xu et al., 2020). VIPS is not suitable for volatile non-solvent systems that are miscible with water. In addition, the breath figure method can be considered as a type of VIPS as their underlying mechanisms are the same.

### 3.3 Electrospinning

Electrospinning is one of the most used technologies for the preparation of fibrous membranes. The main preparation process is to prepare a small amount of spinning liquid and inject it into the spinning machine (Huang and Thomas, 2018). The main principle is to promote the surface of the spinning liquid through the high-voltage electric field to generate current, resulting in stretching and splitting of the spinning liquid and causing the liquid to move along the spiral trajectory to the receiving device and solidify into nanofibers (Khajavi and Abbasipour, 2012). By using this method, a large number of fibers overlapping each other are made to form a porous film (Khajavi and Abbasipour, 2012; Huang and Thomas, 2020). Electrospinning can be combined with phase separation technology to produce nanofibrous films with surface roughness at nanoscale (Rezabeigi and Demarquette, 2019; Huang and Thomas, 2020).

Chen X et al. prepared polycaprolactone (PCL)/PLA core-shell porous drug-carrying nanofibers using coaxial electrospinning technology and non-solvent-induced phase separation technique. Chloroform/DMSO was used as solvent/non-solvent for both the core and the shell layers. In the process of jet flow, chloroform in both layers was first evaporated, generating phase separation with the formation of polymer aggregation areas and non-solvent aggregation areas. Non-solvent DMSO was evaporated after chloroform evaporation, forming holes on the surface of the nanofibers. The nanofibers with pores of different sizes can slow down the drug burst release and increase the dissolution of hydrophobic drugs (Chen et al., 2021a).

### 3.4 Foaming

Foaming process mainly include bubble nucleation, expansion and fixation (Li et al., 2018). Factors affecting PLA foaming include temperature, saturation pressure of the nucleation stage of the foaming, cooling rate of the stabilization stage, tensile viscosity, strength and crystallinity of the polymer melt, the type and amount of the blowing agent, *etc.* (Han et al., 1976; Ding et al., 2016; Tiwary et al., 2017). PLA stereoscopic foam composites have been used to fabricate stable micelles applied to drug delivery and tissue engineering scaffolds (Zhou et al., 2016; Kuang et al., 2017).

Kuang et al. used pressure-induced flow (PIF)-assisted foaming to produce PLLA foams with low-density and high porosity (Kuang et al., 2017). PLLA foams with high-strength, low-density and uniform cellular morphology was produced. Long-term culture of mouse embryonic fibroblast cells (MEFs) demonstrated that the open-cellular PLLA scaffold provided prominent advantages such as enhanced cell adhesion and proliferation and improved nutrient transportation (Kuang et al., 2017).

### 3.5 Double Pickering emulsion

PLA microcapsules, i.e., oil-water-oil (W1/O/W2) systems are prepared by double Pickering microemulsion method. The aqueous phase W) can carry hydrophilic drugs, while PLA is used as an oil phase O) to efficiently embed hydrophobic drugs. For drug-targeted drug release, tissue culture (Zhu et al., 2018). Guo et al. prepared PLA microcapsules by double Pickering emulsion method, using HA and GO as stabilizers for the inner and outer aqueous phases, respectively, the aqueous phase and the oil phase can be loaded with hydrophilic and hydrophobic drugs, respectively, and different layers of drugs can achieve segmented release (Guo et al., 2017). The microcapsular surface promotes initial cell attachment, leading to increased cell activity, while the hollow microcapsule structure allows nutrients and gases to circulate within the structure, making it suitable for cell proliferation, and its cladding structure also facilitates drug-controlled release.

### 3.6 Properties of different self-assembled PLA

In summary, above methods can fabricate PLA materials applied to medical field. BF is a simple and easy method to construct PLA biomaterial with microporous surface structures. But it is difficult to precisely control the size of the micropores (Bormashenko, 2017). Phase separation can produce sponge-like structure suits for cell culture, but it has disadvantages of energy inefficiency and non-suitability for large-scale production (Szewczyk and Stachewicz, 2020; Chen et al., 2021b). Electrospinning technology can industrially produce porous PLA membranes, but the production efficiency is relatively low, the pore size distribution is wide, and the mechanical properties of the fibers are relatively poor (Khorshidi et al., 2016; Lin et al., 2020). Foaming can produce materials with low-density and high porosity, but the foam has loose structure and non-suitability for produce bone scaffold. Double Pickering microemulsion can prepare microcapsules that are able to efficiently embed hydrophobic drugs. The microstructures fabricated above will promote cell attachment and provide anchorage points to the cell, store up the necessary nutrient or medicine and realize the release of loaded medicine.

## 4 Conclusion and future perspectives

The fabrication of biointerfaces that suitable for cellular physiological environments is critical. Materials with interfacial microstructure are required in the biomedical field. Self-assembled PLA with microstructured surfaces can be applied in tissue engineering scaffold, drug-controlled release, tumor therapy, and other biomedical areas. Self-assembling methods including breath figure, phase separation, electrospinning and foaming have showed great potential in fabricating PLA with desired topography for biomedical applications. However, some methods still have issues. For example, PLA scaffolds prepared by phase separation have small pores which hinder cell penetration and prepared by electrospinning have low thermal stability. More research is needed to address these issues. In the future, the research focus will be developing simple and cost-effective fabrication techniques suitable for scale-up and translation to medical market to broaden PLA’s application in biomedical industry.

## References

[B1] Akbarzadeh R. Yousefi A.-M. (2014). Effects of processing parameters in thermally induced phase separation technique on porous architecture of scaffolds for bone tissue engineering. J. Biomed. Mat. Res. B Appl. Biomater. 102, 1304–1315. 10.1002/jbm.b.33101 24425207

[B2] Bormashenko E. (2017). Breath-figure self-assembly, a versatile method of manufacturing membranes and porous structures: Physical, chemical and technological aspects. Membranes 7, 45. 10.3390/membranes7030045 28813026 PMC5618130

[B3] Bui V.-T. Thuy L. T. Tran Q. C. Nguyen V.-T. Dao V.-D. Choi J. S. (2017). Ordered honeycomb biocompatible polymer films via a one-step solution-immersion phase separation used as a scaffold for cell cultures. Chem. Eng. J. 320, 561–569. 10.1016/j.cej.2017.03.086

[B4] Bunz U. H. F. (2006). Breath figures as a dynamic templating method for polymers and nanomaterials. Adv. Mat. 18, 973–989. 10.1002/adma.200501131

[B5] Bysell H. Månsson R. Hansson P. Malmsten M. (2011). Microgels and microcapsules in peptide and protein drug delivery. Adv. Drug Deliv. Rev. 63, 1172–1185. 10.1016/j.addr.2011.08.005 21914455

[B6] Chen X. Li H. Lu W. Guo Y. (2021a). Antibacterial porous coaxial drug-carrying nanofibers for sustained drug-releasing applications. Nanomaterials 11, 1316. 10.3390/nano11051316 34067723 PMC8157037

[B7] Chen Y.-R. Chung H.-W. Tung S.-H. (2021b). On the formation mechanism of nonsolvent-induced porous polylactide electrospun fibers. ACS Appl. Polym. Mat. 3, 5096–5104. 10.1021/acsapm.1c00855

[B8] da Silva D. J. Rosa D. S. (2022). Antimicrobial performance of bioinspired PLA fabricated via one-step plasma etching with silver and copper. ACS Appl. Polym. Mat. 4, 7162–7172. 10.1021/acsapm.2c01043

[B9] Ding W. Jahani D. Chang E. Alemdar A. Park C. B. Sain M. (2016). Development of PLA/cellulosic fiber composite foams using injection molding: Crystallization and foaming behaviors. Compos. Part Appl. Sci. Manuf. 83, 130–139. 10.1016/j.compositesa.2015.10.003

[B10] Duarte A. R. C. Maniglio D. Sousa N. Mano J. F. Reis R. L. Migliaresi C. (2017). From honeycomb- to microsphere-patterned surfaces of poly(lactic acid) and a starch-poly(lactic acid) blend via the breath figure method. J. Appl. Biomater. Funct. Mat. 15, 31–42. 10.5301/jabfm.5000281 27647384

[B11] Ermis M. Antmen E. Hasirci V. (2018). Micro and nanofabrication methods to control cell-substrate interactions and cell behavior: A review from the tissue engineering perspective. Bioact. Mat. 3, 355–369. 10.1016/j.bioactmat.2018.05.005 PMC6026330 29988483

[B12] Ferrari E. Fabbri P. Pilati F. (2011). Solvent and substrate contributions to the formation of breath figure patterns in polystyrene films. Langmuir 27, 1874–1881. 10.1021/la104500j 21226506

[B13] François B. Pitois O. François J. (1995). Polymer films with a self-organized honeycomb morphology. Adv. Mat. 7, 1041–1044. 10.1002/adma.19950071217

[B14] Garcia J. U. Iwama T. Chan E. Y. Tree D. R. Delaney K. T. Fredrickson G. H. (2020). Mechanisms of asymmetric membrane formation in nonsolvent-induced phase separation. ACS Macro Lett. 9, 1617–1624. 10.1021/acsmacrolett.0c00609 35617063

[B15] Guillen G. R. Pan Y. Li M. Hoek E. M. V. (2011). Preparation and characterization of membranes formed by nonsolvent induced phase separation: A review. Ind. Eng. Chem. Res. 50, 3798–3817. 10.1021/ie101928r

[B16] Guo H. Wang Y. Huang Y. Huang F. Li S. Shen Y. (2017). A GO@PLA@HA composite microcapsule: Its preparation and multistage and controlled drug release. Eur. J. Inorg. Chem. 2017, 3312–3321. 10.1002/ejic.201700193

[B17] Han C. D. Kim Y. W. Malhotra K. D. (1976). A study of foam extrusion using a chemical blowing agent. J. Appl. Polym. Sci. 20, 1583–1595. 10.1002/app.1976.070200615

[B18] Han L. Liu H. Fu H. Hu Y. Fang W. Liu J. (2022). Exosome-delivered BMP-2 and polyaspartic acid promotes tendon bone healing in rotator cuff tear via Smad/RUNX2 signaling pathway. Bioengineered 13, 1459–1475. 10.1080/21655979.2021.2019871 35258414 PMC8805918

[B19] Honarbakhsh S. Pourdeyhimi B. (2011). Scaffolds for drug delivery, part I: Electrospun porous poly(lactic acid) and poly(lactic acid)/poly(ethylene oxide) hybrid scaffolds. J. Mat. Sci. 46, 2874–2881. 10.1007/s10853-010-5161-5

[B20] Huang C. Kamra T. Chaudhary S. Shen X. (2014). Breath figure patterns made easy. ACS Appl. Mat. Interfaces 6, 5971–5976. 10.1021/am501096k 24689785

[B21] Huang C. Thomas N. L. (2018). Fabricating porous poly(lactic acid) fibres via electrospinning. Eur. Polym. J. 99, 464–476. 10.1016/j.eurpolymj.2017.12.025

[B22] Huang C. Thomas N. L. (2020). Fabrication of porous fibers via electrospinning: Strategies and applications. Polym. Rev. 60, 595–647. 10.1080/15583724.2019.1688830

[B23] Ismail N. Venault A. Mikkola J.-P. Bouyer D. Drioli E. Tavajohi Hassan Kiadeh N. (2020). Investigating the potential of membranes formed by the vapor induced phase separation process. J. Membr. Sci. 597, 117601. 10.1016/j.memsci.2019.117601

[B24] Jeong E.-G. Yoo H. J. Song B. Kim H.-P. Han S.-W. Kim T.-Y. (2015). Evaluation of lapatinib powder-entrapped biodegradable polymeric microstructures fabricated by X-ray lithography for a targeted and sustained drug delivery system. Materials 8, 519–534. 10.3390/ma8020519 28787954 PMC5455267

[B25] Jin Y. Wang J. Ke H. Wang S. Dai Z. (2013). Graphene oxide modified PLA microcapsules containing gold nanoparticles for ultrasonic/CT bimodal imaging guided photothermal tumor therapy. Biomaterials 34, 4794–4802. 10.1016/j.biomaterials.2013.03.027 23557859

[B26] Kamide K. Iijima H. Matsuda S. (1993). Thermodynamics of formation of porous polymeric membrane by phase separation method I. Nucleation and growth of nuclei. Polym. J. 25, 1113–1131. 10.1295/polymj.25.1113

[B27] Khajavi R. Abbasipour M. (2012). Electrospinning as a versatile method for fabricating coreshell, hollow and porous nanofibers. Sci. Iran. 19, 2029–2034. 10.1016/j.scient.2012.10.037

[B28] Khorshidi S. Solouk A. Mirzadeh H. Mazinani S. Lagaron J. M. Sharifi S. (2016). A review of key challenges of electrospun scaffolds for tissue-engineering applications. J. Tissue Eng. Regen. Med. 10, 715–738. 10.1002/term.1978 25619820

[B29] Kim J. F. Kim J. H. Lee Y. M. Drioli E. (2016). Thermally induced phase separation and electrospinning methods for emerging membrane applications: A review. AIChE J. 62, 461–490. 10.1002/aic.15076

[B30] Kuang T. Chen F. Chang L. Zhao Y. Fu D. Gong X. (2017). Facile preparation of open-cellular porous poly (l-lactic acid) scaffold by supercritical carbon dioxide foaming for potential tissue engineering applications. Chem. Eng. J. 307, 1017–1025. 10.1016/j.cej.2016.09.023

[B31] Lassalle V. Ferreira M. L. (2007). PLA nano- and microparticles for drug delivery: An overview of the methods of preparation. Macromol. Biosci. 7, 767–783. 10.1002/mabi.200700022 17541922

[B32] Li B. Zhao G. Wang G. Zhang L. Gong J. (2018). Fabrication of high-expansion microcellular PLA foams based on pre-isothermal cold crystallization and supercritical CO2 foaming. Polym. Degrad. Stab. 156, 75–88. 10.1016/j.polymdegradstab.2018.08.009

[B33] Lin W. Chen M. Qu T. Li J. Man Y. (2020). Three-dimensional electrospun nanofibrous scaffolds for bone tissue engineering. J. Biomed. Mat. Res. B Appl. Biomater. 108, 1311–1321. 10.1002/jbm.b.34479 31436374

[B34] Liu S. Qin S. He M. Zhou D. Qin Q. Wang H. (2020). Current applications of poly(lactic acid) composites in tissue engineering and drug delivery. Compos. Part B Eng. 199, 108238. 10.1016/j.compositesb.2020.108238

[B35] Mansouri J. Yapit E. Chen V. (2013). Polysulfone filtration membranes with isoporous structures prepared by a combination of dip-coating and breath figure approach. J. Membr. Sci. 444, 237–251. 10.1016/j.memsci.2013.05.022

[B36] Mi H.-Y. Salick M. R. Jing X. Jacques B. R. Crone W. C. Peng X.-F. (2013). Characterization of thermoplastic polyurethane/polylactic acid (TPU/PLA) tissue engineering scaffolds fabricated by microcellular injection molding. Mat. Sci. Eng. C 33, 4767–4776. 10.1016/j.msec.2013.07.037 PMC4554542 24094186

[B37] Ozaltin K. Vargun E. Di Martino A. Capakova Z. Lehocky M. Humpolicek P. (2022). Cell response to PLA scaffolds functionalized with various seaweed polysaccharides. Int. J. Polym. Mat. Polym. Biomater. 71, 79–86. 10.1080/00914037.2020.1798443

[B38] Pitois O. François B. (1999). Crystallization of condensation droplets on a liquid surface. Colloid Polym. Sci. 277, 574–578. 10.1007/s003960050427

[B39] Qi W. Zhang X. Wang H. (2018). Self-assembled polymer nanocomposites for biomedical application. Curr. Opin. Colloid Interface Sci. 35, 36–41. 10.1016/j.cocis.2018.01.003

[B40] Rezabeigi E. Demarquette N. R. (2019). Ultraporous membranes electrospun from nonsolvent-induced phase-separated ternary systems. Macromol. Rapid Commun. 40, 1800880. 10.1002/marc.201800880 30747462

[B41] Riley T. Heald C. R. Stolnik S. Garnett M. C. Illum L. Davis S. S. (2003). Core−Shell structure of PLA−PEG nanoparticles used for drug delivery. Langmuir 19, 8428–8435. 10.1021/la020911h

[B42] Shah Mohammadi M. Bureau M. N. Nazhat S. N. (2014). “11 - polylactic acid (PLA) biomedical foams for tissue engineering,” in Biomedical foams for tissue engineering applications. Editor Netti P. A. (Canada: McGill University, Woodhead Publishing), 313–334. 10.1533/9780857097033.2.313

[B43] Stenzel M. H. Barner-Kowollik C. Davis T. P. (2006). Formation of honeycomb-structured, porous films via breath figures with different polymer architectures. J. Polym. Sci. Part Polym. Chem. 44, 2363–2375. 10.1002/pola.21334

[B44] Sun L. Danoux C. B. Wang Q. Pereira D. Barata D. Zhang J. (2016). Independent effects of the chemical and microstructural surface properties of polymer/ceramic composites on proliferation and osteogenic differentiation of human MSCs. Acta Biomater. 42, 364–377. 10.1016/j.actbio.2016.06.018 27318269

[B45] Szewczyk P. K. Stachewicz U. (2020). The impact of relative humidity on electrospun polymer fibers: From structural changes to fiber morphology. Adv. Colloid Interface Sci. 286, 102315. 10.1016/j.cis.2020.102315 33197707

[B46] Tiwary P. Park C. B. Kontopoulou M. (2017). Transition from microcellular to nanocellular PLA foams by controlling viscosity, branching and crystallization. Eur. Polym. J. 91, 283–296. 10.1016/j.eurpolymj.2017.04.010

[B47] van de Witte P. Dijkstra P. J. van den Berg J. W. A. Feijen J. (1996). Phase separation processes in polymer solutions in relation to membrane formation. J. Membr. Sci. 117, 1–31. 10.1016/0376-7388(96)00088-9

[B48] van Kooten T. G. Spijker H. T. Busscher H. J. (2004). Plasma-treated polystyrene surfaces: Model surfaces for studying cell–biomaterial interactions. Biomaterials 25, 1735–1747. 10.1016/j.biomaterials.2003.08.071 14738836

[B49] Wang D.-M. Lai J.-Y. (2013). Recent advances in preparation and morphology control of polymeric membranes formed by nonsolvent induced phase separation. Curr. Opin. Chem. Eng. 2, 229–237. 10.1016/j.coche.2013.04.003

[B50] Wang F. Guo G. Ma Q. Gu M. Wu X. Sheng S. (2013). Investigation on the thermo-mechanical properties and thermal stability of polylactic acid tissue engineering scaffold material. J. Therm. Anal. Calorim. 113, 1113–1121. 10.1007/s10973-013-3221-1

[B51] Wang Z. Pan Z. Wang J. Zhao R. (2016). A novel hierarchical structured poly(lactic acid)/titania fibrous membrane with excellent antibacterial activity and air filtration performance. J. Nanomater. 39, 1–17. 10.1155/2016/6272983

[B52] Weiss P. (1968). “The problem of specificity in growth and development,” in Dynamics of development: Experiments and inferences (Elsevier), 265–308. 10.1016/B978-1-4832-2919-5.50016-7

[B53] Widawski G. Rawiso M. François B. (1994). Self-organized honeycomb morphology of star-polymer polystyrene films. Nature 369, 387–389. 10.1038/369387a0

[B54] Wu D. Spanou A. Diez-Escudero A. Persson C. (2020). 3D-printed PLA/HA composite structures as synthetic trabecular bone: A feasibility study using fused deposition modeling. J. Mech. Behav. Biomed. Mat. 103, 103608. 10.1016/j.jmbbm.2019.103608 32090935

[B55] Wu X. H. Wu Z. Y. Su J. C. Yan Y. G. Yu B. Q. Wei J. (2014). Nano-hydroxyapatite promotes self-assembly of honeycomb pores in poly(L-lactide) films through breath-figure method and MC3T3-E1 cell functions. RSC Adv. 5, 6607–6616. 10.1039/C4RA13843K

[B56] Xiong X. Zou W. Yu Z. Duan J. Liu X. Fan S. (2009). Microsphere pattern prepared by a “reverse” breath figure method. Macromolecules 42, 9351–9356. 10.1021/ma9018119

[B57] Xu M.-H. Xie R. Ju X.-J. Wang W. Liu Z. Chu L.-Y. (2020). Antifouling membranes with bi-continuous porous structures and high fluxes prepared by vapor-induced phase separation. J. Membr. Sci. 611, 118256. 10.1016/j.memsci.2020.118256

[B58] Yin H. Zhan F. Yu Y. Li Z. Feng Y. Billon L. (2019). Direct formation of hydrophilic honeycomb film by self-assembly in breath figure templating of hydrophobic polylacticacid/ionic surfactant complexes. Soft Matter 15, 5052–5059. 10.1039/C9SM00845D 31180399

[B59] Zhang A. Bai H. Li L. (2015). Breath figure: A nature-inspired preparation method for ordered porous films. Chem. Rev. 115, 9801–9868. 10.1021/acs.chemrev.5b00069 26284609

[B60] Zhou C. Yang K. Wang K. Pei X. Dong Z. Hong Y. (2016). Combination of fused deposition modeling and gas foaming technique to fabricated hierarchical macro/microporous polymer scaffolds. Mat. Des. 109, 415–424. 10.1016/j.matdes.2016.07.094

[B61] Zhu J.-Y. Tang C.-H. Yin S.-W. Yang X.-Q. (2018). Development and characterization of novel antimicrobial bilayer films based on Polylactic acid (PLA)/Pickering emulsions. Carbohydr. Polym. 181, 727–735. 10.1016/j.carbpol.2017.11.085 29254029

